# Biochemical Signals Mediate the Crosstalk between Cartilage and Bone in Osteoarthritis

**DOI:** 10.1155/2020/5720360

**Published:** 2020-04-06

**Authors:** Xuchang Zhou, Hong Cao, Yu Yuan, Wei Wu

**Affiliations:** ^1^School of Kinesiology, Shanghai University of Sport, Shanghai, China; ^2^School of Physical Education and Sports Science, South China Normal University, Guangzhou, China

## Abstract

Osteochondral junction is a functional unit comprising the articular cartilage, calcified cartilage, and subchondral bone. Alteration in any component of this composite unit can disrupt the joint integrity and function directly or indirectly. Biochemical signals mediate the crosstalk between tissues and play an essential role in the initiation and progression of osteoarthritis. As osteoarthritis progresses, abnormal subchondral bone remodelling leads to increased angiogenesis and porosity of the subchondral bone plate, which further triggers biochemical signals to mediate the crosstalk between cartilage and bone, contributing to the progression of osteoarthritis. Notably, common biochemical signals include the TGF-*β*/Smad, Wnt/*β*-catenin, RANK/RANKL/OPG, and MAPK pathways. This biomarker crosstalk network is the basis of osteoarthritis pathogenesis, and some of their key regulators may be potential therapeutic targets for osteoarthritis drug therapy. This review summarised the biochemical crosstalk between cartilage and bone in the pathogenesis of osteoarthritis, which may provide the basis for the discovery of osteoarthritis treatment targets.

## 1. Introduction

Osteoarthritis (OA) is widely accepted as a common degenerative joint disease affected by biomechanics and biochemical signals, involving the entire joint, including the overlying articular cartilage (AC), the calcified cartilage (CC), and the underlying subchondral bone (SB). The occurrence of OA is related to heredity, age, sex, weight, joint trauma, and abnormal mechanical loading. OA is characterised by various phenotypes such as cartilage degeneration and calcification, synovial inflammation, osteophyte formation, vascular invasion, subchondral remodelling, and increased porosity in the subchondral bone plate (SBP) [1, 2]. Although numerous studies have been conducted on the pathogenesis and treatment of OA, there are no effective strategies to reverse or block the progressive destruction of cartilage and periarticular bone in OA [3].

It is believed that the main causes of OA are chondrocyte apoptosis and matrix degeneration [4]. Radin and Rose [5] first clarified that damage to SB could lead to the degeneration of AC, indicating that the abnormality of SB may contribute to the progression of AC damage. Subsequently, numerous studies have suggested that in OA, the morphological changes of periarticular bone, such as bone remodelling and osteophyte formation, may occur earlier than the changes in AC [6, 7]. The asynchronous changes in bone and cartilage may be due to various factors involved in the initiation of OA, and different pathogenesis results in various degrees of SB lesions at different times.

Furthermore, recent studies have shown that there is molecular crosstalk between cartilage and SB contributing to OA pathology by increasing microcracks, vascular channels, and porosity of SB [8–10]. Therefore, a better understanding of the crosstalk between tissues in the osteochondral junction may help to develop strategies that are more effective in treating OA. This review focuses on the biochemical signals that mediate the crosstalk in the osteochondral unit to provide the basis for more effective therapeutic targets and strategies to treat OA.

## 2. The Cartilage-Bone Unit

### 2.1. Articular Cartilage

Articular cartilage (AC) lacks blood vessels and nerve fibers and is composed of 1% chondrocyte and 99% extracellular matrix [11]. In daily life, AC is under compression, shear, and other complex mechanical stimuli. Chondrocytes quickly respond to changes in the AC microenvironment and secrete proteoglycan, COL-II, MMPs, and other components to adjust the dynamic balance between degradation and synthesis of the extracellular matrix, which plays a crucial role in maintaining the cartilage homeostasis [12]. When mechanical stress is beyond the daily loading, chondrocytes lose their resting phenotype, resulting in an abnormal hypertrophy and proliferation, reduction in activity, and synthesis of many MMPs, aggrecanases, proteoglycan, and COL-X by chondrocytes, resulting in chondrocyte metabolism disorders [13].

### 2.2. Calcified Cartilage

Calcified cartilage (CC) is made up of 20% COL-II (40% less than the COL-II in AC) and 65% low crystalline hydroxyapatite (20% less than the low crystalline hydroxyapatite in AC), and it exists between the overlying AC and the underlying SB [14]. CC is a highly mineralised matrix zone comprising proteoglycan, collagen fibers, and hydroxyapatite, functioning as a barrier between AC and SB. Moreover, it prevents O_2_ from passing through the CC layer, resulting in a special hypoxic microenvironment of the cartilage tissue. Meanwhile, the semipermeable membrane effect of CC maintains the joint homeostasis by providing channels for small molecules [15]. However, with the progression of OA, the barrier function of CC is disrupted, leading to an abnormal biochemical crosstalk in the osteochondral junction to promote microcracks and angiogenesis, thus aggravating AC [16, 17].

### 2.3. Subchondral Bone

Subchondral bone (SB) consists of SBP and subchondral trabecular bone. SB acts as a cushioning structure in response to a mechanical loading, attenuating mechanical loading in the entire joint by approximately 30%, compared to that attenuated by the AC, which only buffers 1%-3% of the mechanical loading [18]. Under physiological conditions, SBP acts as a barrier layer in the osteochondral junction to maintain an AC homeostasis and integrity, blocks both vessels and nerves from SB into AC, and ensures that the bone tissue fluid penetrates the barrier layer, thereby nourishing the deep layer of AC [19]. However, an uncoupled bone remodelling occurs in the SB during the progression of OA, leading to an increase in the SBP porosity, accompanied by the appearance of microcracks and vascular invasion. This allows some molecules to pass through SBP to AC accompanied by an SB deterioration and AC degradation, contributing to the initiation and progression of OA [8, 20].

## 3. Biochemical Crosstalk in the Cartilage-Bone Unit

Many studies directly or indirectly suggest that there are complex biochemical signals that mediate the crosstalk of cartilage bone to regulate the progression of OA. Compared to the normal subchondral osteoblasts in vitro, a higher level of osteogenic biomarkers such as alkaline phosphatase (ALP) can be seen in these OA-affected osteoblasts [21]. The factors secreted by osteoblasts in a sclerotic bone contribute to the decreased level of COL-II and increased levels of the matrix metalloproteinase- (MMP-) 3 and MMP-13 in normal chondrocytes which cannot be secreted by normal osteoblasts [22, 23]. In contrast, the factors secreted by OA chondrocytes can enhance the activities of MMP-1 and MMP-2 in both normal and OA subchondral osteoblasts [24]. In OA rats induced by surgery in vivo, the porosity in the SB and AC degeneration is consistent with those in the area of the knee that undergoes a mechanical loading, and the AC degeneration and adjacent SB damage are time-dependent [8]. Moreover, an increase in the SB turnover in early OA may lead to changes in the mechanical transmission and a progressive AC loss [25]. Evidence has shown that OA progression can be reduced using drugs that inhibit the bone turnover [26]. Further, human epidemiological studies have confirmed that the progression of knee OA is related to an increase in the SB turnover [27]. As described, there is evidence from in vitro, in vivo, and clinical studies that the communication between cartilage and bone can promote OA pathogenesis. It is suggested that the study of biochemical signals in the cartilage bone unit may contribute to the discovery of new strategies for OA treatment.

## 4. The TGF-*β*/Smad Signalling Pathway

TGF-*β* signalling regulates the bone and chondrocyte metabolism under physiological and pathological conditions, depending on specific membrane receptors (type I receptors are known as activin receptor-like kinases (ALKs) and type II receptors are known as TGF-*β*RII), and subsequently activates its intracellular effectors and the phosphorylation of Smad proteins [28]. Evidence shows that TGF-*β* is highly expressed in the normal AC but hardly expressed in an OA AC [29]. Inhibition of the TGF-*β*/Smad signalling pathway deteriorates cartilage degeneration, and activation of the TGF-*β*/Smad signalling pathway attenuates cartilage damage [30]. Accordingly, TGF-*β* may play an essential role in mediating the biochemical crosstalk of the cartilage bone unit and in maintaining the metabolic balance and structural integrity of cartilage in OA.

Chondrocyte metabolic activity depends on the balance of ALK1 and ALK5 expression in response to TGF-*β* stimulation: the ALK1-induced Smad1/5/8 pathway is associated with catabolism by upregulating MMP-13 [31]; the ALK5-induced Smad2/3 signalling is associated with anabolism by upregulating aggrecan and COL-II and inhibiting MMP-13 and COL10A1 [32]. During the progression of OA, SB undergoes an uncoupled bone remodelling due to bone formation at wrong sites caused by an excessive release and activation of TGF-*β* in SB under an abnormal mechanical loading [6]. Enhanced bone remodelling increases the porosity of SBP and vascular channels, allowing the stromal cell-derived factor-1 (SDF-1) to transform from SB to AC in combination with the C-X-C chemokine receptor type 4 (CXCR4). This subsequently leads to an upregulation in chondrocytes in the ratio of ALK1/ALK5: the ALK5-mediated expression of protective Smad2/3 is reduced; the ALK1-mediated Smad1/5/8 induces cartilage matrix degradation, ultimately leading to cartilage deterioration [33]. In brief, TGF-*β* signalling mediates an osteochondral biochemical crosstalk by increasing the porosity of SBP and vascular channels and participating in the regulation of osteochondral metabolism.

## 5. The Wnt/*β*-Catenin Signalling Pathway

Wnt/*β*-catenin signalling has an opposite dual effect during OA progression. Appropriate levels of *β*-catenin are essential for cartilage homeostasis. Inhibition or an overexpression of *β*-catenin signalling perturbs the chondrocyte phenotype, promoting chondrocyte apoptosis and cartilage destruction [34, 35]. Moreover, an increased expression of Wnt signalling agonists in the human OA cartilage may accelerate cartilage degradation by upregulating MMPs and aggrecanases [36]. In addition to regulating chondrocyte survival, the Wnt/*β*-catenin signalling pathway is involved in skeletal development and homeostasis by regulating osteoblast and osteoclast functions. Studies have shown that a proper mechanical loading promotes bone formation and increases the SB thickness and trabecular volume fraction by activating Wnt/*β*-catenin signalling. Moreover, inhibition of osteoclastogenesis and osteoclast activity regulates SB reconstruction and improves the abnormal bone microstructure [37, 38]. It can be concluded that the regulation of Wnt/*β*-catenin signalling is a crucial event in cartilage and bone metabolism.

There is an interaction between the Wnt/*β*-catenin and TGF-*β*/Smad signalling pathways, in which the Wnt-induced secreted protein-1 (WISP1) is the key regulator. WISP1 regulates the initiation and progression of OA by acting on multiple levels of lesions as follows: (1) accelerating the release of MMPs and proteoglycans, subsequently leading to the destruction of the cartilage matrix; (2) promoting the conversion of chondrocytes to a hypertrophic phenotype by inducing the cartilage-destroying Smad1/5/8 signalling pathway; and (3) stimulating SB sclerosis and osteophyte formation by regulating osteogenesis and osteoclast function [39]. This implies that there may be a complex interaction between the Wnt/*β*-catenin and TGF-*β*/Smad signalling pathways to jointly regulate the homeostasis of the whole joint in OA pathology.

## 6. The RANK/RANKL/OPG Signalling Pathway

The prominent members of the receptor activator of the NF-*κ*B (RANK)/receptor activator of NF-*κ*B ligand (RANKL)/osteoprotegerin (OPG) signalling pathway include RANK, RANKL, and OPG. In OA progression, RANKL secreted by the chondrocytes may not be involved in chondrocyte activation or cartilage degradation, as exogenous RANKL does not trigger NF-*κ*B activation or the transcription of proinflammatory cytokine genes [40]. However, RANKL diffuses from AC through CC into SB and participates in the regulation of SB remodelling. Furthermore, the metabolic activity of OA osteoblasts is higher than that of normal osteoblasts, which have lower RANKL levels and higher OPG levels. OPG secreted by osteoblasts acts as a decoy receptor in the form of a dimer to competitively bind to the trimeric RANKL owing to its high affinity, which causes RANKL to lose its binding to RANK, blocking the interaction between RANKL and RANK and effectively inhibiting the differentiation, activation, and maturation of osteoclasts, resulting in decreased bone resorption [40]. The ratio of RANKL/OPG has been reported to increase first and then decrease in OA [40], which is consistent with the pathological changes in which bone resorption is dominant in the early stage of OA and bone formation is dominant in the late stage. In short, the RANK/RANKL/OPG signalling participates in the regulation of bone remodelling through an osteochondral crosstalk at different stages of an OA pathological process.

### 6.1. The RANK/RANKL/OPG Signalling Pathway Regulates Osteoclastogenesis with TGF-*β* Signalling

Osteoclasts are affected by various proinflammatory osteoclastogenic and antiosteoclastogenic cytokines [41]. Studies have shown that antiosteoclastogenic cytokines, such as interferon-gamma (IFN-*γ*), significantly inhibit osteoclastogenesis by inhibiting RANK signalling [42, 43]. TGF-*β*1 is involved in breaking the balance of bone resorption and bone formation homeostasis by releasing inflammatory factors in the pathological bone microenvironment [44]. Inflammatory factors such as IL-1*α*, IL-6, and TNF-*α* can directly act on specific receptors of osteoclasts or indirectly on osteoclasts through the RANK/RANKL/OPG signalling pathway to induce osteoclastogenesis [45, 46]. Studies have shown that in the absence of inflammation and oestrogen, the expression of RANKL is significantly increased, whereas the expression of OPG is decreased and antiosteogenesis of IFN-*γ* is inhibited [47, 48]. Briefly, TGF-*β*1 inhibits the antiosteogenesis of IFN-*γ* and induces the expression of osteoclastogenesis genes by releasing inflammatory factors and the RANK/RANKL/OPG pathway, which ultimately promotes osteoclastogenesis.

### 6.2. The RANK/RANKL/OPG Signalling Pathway Regulates the Subchondral Bone Remodelling with the Wnt/*β*-Catenin Signalling Pathway

The RANK/RANKL/OPG signalling pathway interacts with the Wnt/*β*-catenin signalling pathway to regulate bone remodelling in the SB. Moreover, in chondrocytes, the OPG/RANKL signalling pathway is involved in *β*-catenin secretion by chondrocytes to regulate the activity of subchondral osteoclasts through osteochondral crosstalk channels, contributing to bone remodelling [49]. Furthermore, in osteoblasts, OPG expression is regulated by the Wnt/*β*-catenin signalling pathway [50]. The knockdown of RUNX2, a crucial regulator of bone formation, induces a high expression of RANKL and inhibits OPG expression, while an overexpression of RUNX2 inhibits the function of the canonical Wnt/*β*-catenin signalling pathway by depletion of *β*-catenin, resulting in a decreased bone mass and volume. However, activation of *β*-catenin reverses high bone resorption of SB in mice due to the overexpression of RUNX2, which is closely related to RANKL/OPG signalling [51]. To summarise, crosstalk between RANK/RANKL/OPG signalling and Wnt/*β*-catenin signalling is an essential part of the regulatory network in SB remodelling.

## 7. MAPK Signalling Pathways

The mitogen-activated protein kinase (MAPK) is a highly conserved serine/threonine-protein kinase family that regulates cellular biological processes by transmitting extracellular signals to the nucleus via phosphorylation cascades. The MAPK signalling pathways, including the c-Jun N-terminal kinase (JNK), p38 MAPK, and extracellular signal-regulated kinase (ERK)1/2, are involved in the pathogenesis of OA. It has been confirmed that both the JNK and p38 MAPK signalling pathways are associated with an apoptosis of the OA chondrocytes [52], while the p38 MAPK and ERK1/2 signalling pathways are closely related to an osteochondral crosstalk [53, 54].

### 7.1. MAPK Signalling Pathways Mediate an Osteochondral Crosstalk through Metabolism Regulatory Factors

Increasing evidence shows that the activation of MAPK, particularly ERK1/2, contributes to the expression of MMPs in chondrocytes [55] and osteoblasts [56]. MMPs, such as MMP-1, MMP-2, MMP-8, MMP-9, and MMP-13, play a crucial role in OA cartilage degradation due to their abnormal expression, which can cleave the collagen triple helix domain, including COL-II [57]. Majumdar et al. [58] showed that the double knockout of ADAMTS-4 and ADAMTS-5 prevented the progression of OA. Signals from OA subchondral osteoblasts in vitro stimulate the expression of ADAMTS-4, ADAMTS-5, MMP-2, MMP-3, and MMP-9 in chondrocytes, whereas OA chondrocytes increase the activities of MMP-1 and MMP-2 in osteoblasts, which is mediated by phosphorylating the ERK1/2 signalling pathway [24], indicating that there may be a catabolism-related bidirectional crosstalk in the osteochondral junction during OA progression. Sanchez et al. [22] designed a coculture model demonstrating that OA subchondral osteoblasts inhibited a normal chondrocyte anabolism, as evidenced by a reduced aggrecan synthesis followed by matrix mineralisation, which may be mediated through both phosphorylation of ERK1/2 signalling and inactivation of p38 phosphorylation [54]. In brief, OA subchondral osteoblasts simultaneously inhibit chondrocyte anabolism and promote chondrocyte catabolism through the ERK1/2 signalling pathway. Therefore, it can be concluded that the MAPK signalling pathways contribute to cartilage and SB damage through the metabolism of regulatory factor crosstalk in the cartilage bone unit.

### 7.2. MAPK Signalling Pathways Mediate an Osteochondral Crosstalk by Regulating Subchondral Bone Formation

The *core binding factor alpha 1* (*CBFA1*) mediates osteoblast differentiation and increases the matrix mineralisation by regulating the expression of osteogenic genes, like *ALP* and *osteocalcin* (*OC*) [59]. Compared to the normal subchondral osteoblasts, OA subchondral osteoblasts considerably express *CBFA1*, *ALP*, and *OC* mRNA, showing higher osteogenic differentiation [22, 53, 60]. ERK1/2 phosphorylation was not observed in the normal chondrocytes cocultured with normal subchondral osteoblasts, but ERK1/2 phosphorylation was activated and *CBFA1*, *ALP*, and *OC* expressions were significantly upregulated in osteoblasts. Moreover, matrix mineralisation was observed when the OA chondrocytes were cocultured with normal subchondral osteoblasts, indicating that OA chondrocytes could contribute to SB formation and subsequent sclerosis by activating the ERK-CBFA1-OC pathway. Furthermore, controversy remains on the participation of p38 phosphorylation in the abovementioned process [53].

## 8. Interaction between Various Signalling Pathways in the Subchondral Bone Remodelling during OA Progression

Bone remodelling of SB relies on the spatiotemporal coupling of an osteoclast-mediated bone resorption and an osteoblast-mediated bone formation. Coupling bone reconstruction ensures the integrity of SB, which is essential for maintaining the SB homeostasis [61]. During OA progression, abnormal mechanical loading leads to changes in the SB microenvironment and bone resorption function is rapidly enhanced. Specifically, osteoclasts secrete hydrochloric acid and proteases (mainly H^+^, Cl^−^, and the cysteine protease cathepsin K), leading to an excessive release and activation of TGF-*β* from the SB matrix [62].

Excessive TGF-*β* promotes osteoclastogenesis. TGF-*β* plays a vital role in initiating the differentiation of the CD4^+^ T cell subsets into the Th17 cell subsets, which induces the expression of RANKL [63, 64]. RANKL is first expressed on the surface of osteoblasts and binds to its receptor RANK to induce trimerisation and activation of TRAF6 on the surface of preosteocytes. Subsequently, a series of downstream signalling cascades, such as the NF-*κ*B and MAPK pathways, are activated to initiate osteoclast differentiation [65], resulting in an enhanced bone resorption and an increased porosity of SBP and vascular channels.

Furthermore, activation of the MAPK signalling pathway leads to an enhanced osteoblast anabolism to promote bone formation. Robinson et al. [66] found that the MAPK signalling pathways synergistically promoted the expression of osteogenic genes associated with the canonical Wnt/*β*-catenin signalling pathway under an appropriate mechanical loading. In contrast, NF-*κ*B could inhibit osteogenic differentiation of the bone marrow mesenchymal stem cells (BMSCs) to negatively regulate the Wnt/*β*-catenin signalling pathway by promoting *β*-catenin degradation [67, 68].

In contrast, an excessive TGF-*β* level promotes an ectopic bone formation in SB. Under an abnormal mechanical loading, excessive TGF-*β* disrupts the coupled bone remodelling of SB and results in an osteoid islet formation in SB marrow by erroneously recruiting BMSCs, which further differentiate into osterix^+^ osteoprogenitors in bone marrow, rather than bone resorption pits on the bone surface [69].

## 9. The Potential Therapeutic Strategies Based on the Biochemical Crosstalk in OA

A successful drug treatment strategy should be able to affect multiple levels of lesions simultaneously in OA [7, 70, 71]. Biochemical crosstalk in the osteochondral junction is a core event in the initiation and progression of OA. Currently, many biomarkers show great potential as targets for OA treatment, such as TGF-*β* and RANKL. Li et al. [72] suggested that artesunate alleviated the excessive release of TGF-*β*, thereby reducing the number of nestin^+^ MSCs, while promoting the transfer of osterix^+^ osteoprogenitors from the bone marrow to the bone surface to inhibit the TGF-*β*/Smad2/3 signalling, ultimately inhibiting an ectopic bone formation. Moreover, Ji et al. [70] showed that isoliquiritigenin both directly inhibited osteoclastogenesis induced by the RANKL-RANK-TRAF6 signalling and indirectly inhibited the excessive release of TGF-*β* to block a series of downstream signalling cascades and alleviate an abnormal bone formation in SB. Besides, Zhu et al. [7] found that alendronate also impeded osteoclastogenesis to protect against cartilage damage by upregulating the OPG/RANKL ratio and inhibiting chondrocyte catabolic factors in ovariectomised OA mice.

## 10. Conclusions

**Figure 1:**
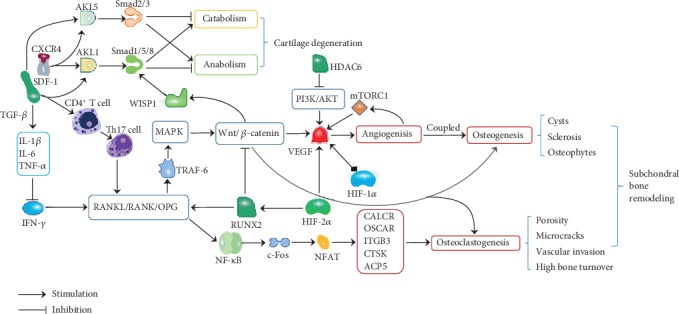
Potential mechanisms underlying osteochondral crosstalk in OA.

OA is a multifactorial degenerative whole joint disease that results in a complex pathogenesis. The initial lesions of OA may be located in AC or SB and interact with each other through biochemical crosstalk between tissues in the osteochondral junction. Abnormal mechanical microenvironment leads to a high bone turnover during uncoupled SB remodelling and promotes the porosity of SBP, microcracks, and angiogenesis, destroying the barrier between AC and SB and increasing the exchange of various cytokines. This biochemical molecular crosstalk contributes to the initiation and progression of OA, and its specific mechanism is complex, involving multiple signalling pathways, including the TGF-*β*/Smad, Wnt/*β*-catenin, RANK/RANKL/OPG, and MAPK signalling pathways (Figure 1). More studies are required to further understand the regulatory network involved in osteochondral crosstalk, providing the basis for more effective therapeutic targets.
